# Supplementary material for: Methylome-based cell-of-origin modeling (Methyl-COOM) identifies aberrant expression of immune regulatory molecules in CLL
Source: Genome Med. 2020 Mar 18;12:29. doi: 10.1186/s13073-020-00724-7 (PMC7081711; doi:10.1186/s13073-020-00724-7)
Supplement: Supplementary file 11 — List of datasets used in the article. [file 13073_2020_724_MOESM11_ESM.docx]

| **Dataset** | **Source**  [**AQ8**](https://eproofing.springer.com/journals_v2/mainpage.php?token=pAOuYMgk7cvzjQoo5i08c6s--QuHSg3UX5XJOENiZpg7NWnONlRUjw) |
| --- | --- |
| Illumina 450 data, normal B cells | Oakes et al. [4] |
| Illumina 450 data, CLLs discovery cohort | Dietrich et al. [21] |
| Illumina 450 data, CLLs validation cohort | Oakes et al. [4] |
| RNAseq, CLLs | Dietrich et al. [21] |
| RNAseq, normal B cells | International Cancer Genome Consortium (ICGC); EGAD00001000258 |
| sncRNAseq, CLL | Blume et al. [20] |
| ENCODE TF ChIP-seq GM12878 | ENCODE project [90] |
| ATAC-seq, normal B cells and CLLs | DKFZ PRECiSE consortium [26] |
| ChIP-seq, normal B cells and CLLs | DKFZ PRECiSE consortium [26]; EGAS00001002518 |
| Promoter segmentation data CLL | DKFZ PRECiSE consortium [26]; GSE113336 |
| ChromHMM GM12878 data | ENCODE ENCSR212BHV [90] |

# Supplementary file 11: List of datasets used in the article

**References**

4. Oakes CC, Seifert M, Assenov Y, Gu L, Przekopowitz M, Ruppert AS, et al. DNA methylation dynamics during B cell maturation underlie a continuum of disease phenotypes in chronic lymphocytic leukemia. Nat Genet. 2016;48:253–64.

20. Blume CJ, Hotz-Wagenblatt A, Hullein J, Sellner L, Jethwa A, Stolz T, et al. p53-dependent non-coding RNA networks in chronic lymphocytic leukemia. Leukemia. 2015;29:2015–23.

21. Dietrich S, Oles M, Lu J, Sellner L, Anders S, Velten B, et al. Drug-perturbation-based stratification of blood cancer. J Clin Invest. 2018;128:427–45.

26. DKFZPRECiSE consortium. DKFZ PRECiSE consortium data resources 2018.

90. Davis CA, Hitz BC, Sloan CA, Chan ET, Davidson JM, Gabdank I, et al. The Encyclopedia of DNA elements (ENCODE): data portal update. Nucleic Acids Res. 2018;46:D794–801.
